# Optical Properties of Silicon Nanowires Fabricated by Environment-Friendly Chemistry

**DOI:** 10.1186/s11671-016-1568-5

**Published:** 2016-08-09

**Authors:** Kirill A. Gonchar, Alsu A. Zubairova, Alexander Schleusener, Liubov A. Osminkina, Vladimir Sivakov

**Affiliations:** 1 Physics Department, Lomonosov Moscow State University, Leninskie Gory 1, 119991 Moscow, Russia; 2 Ural Federal University, 19 Mira Street, 620002 Yekaterinburg, Russia; 3 Leibniz Institute of Photonic Technology, Albert-Einstein Street 9, 07745 Jena, Germany; 4 National Research Nuclear University “MEPhI” (Moscow Engineering Physics Institute), 31 Kashirskoe sh., 115409 Moscow, Russia

**Keywords:** Silicon nanowires, Environment-friendly chemistry, Total reflectance, Photoluminescence, Raman scattering

## Abstract

Silicon nanowires (SiNWs) were fabricated by metal-assisted chemical etching (MACE) where hydrofluoric acid (HF), which is typically used in this method, was changed into ammonium fluoride (NH_4_F). The structure and optical properties of the obtained SiNWs were investigated in details. The length of the SiNW arrays is about 2 μm for 5 min of etching, and the mean diameter of the SiNWs is between 50 and 200 nm. The formed SiNWs demonstrate a strong decrease of the total reflectance near 5–15 % in the spectral region *λ* < 1 μm in comparison to crystalline silicon (c-Si) substrate. The interband photoluminescence (PL) and Raman scattering intensities increase strongly for SiNWs in comparison with the corresponding values of the c-Si substrate. These effects can be interpreted as an increase of the excitation intensity of SiNWs due to the strong light scattering and the partial light localization in an inhomogeneous optical medium. Along with the interband PL was also detected the PL of SiNWs in the spectral region of 500–1100 nm with a maximum at 750 nm, which can be explained by the radiative recombination of excitons in small Si nanocrystals at nanowire sidewalls in terms of a quantum confinement model. So SiNWs, which are fabricated by environment-friendly chemistry, have a great potential for use in photovoltaic and photonics applications.

## Background

In the last years, silicon nanowires (SiNWs) are of great interest because of their potential applications in micro- and optoelectronics [1, 2], photonics [3], photovoltaics [4, 5], and sensorics [6–8]. Initially, SiNWs were obtained by vapor-liquid-solid method with the help of a noble metal (mostly gold, Au) which was first proposed by Wagner and Ellis in 1964 [9]. An alternative method was metal-assisted chemical etching (MACE) by which initially it was believed that the porous silicon (PSi) was obtained [10, 11]. MACE is based on the anisotropic etching of crystalline silicon (c-Si) in aqueous solutions which are usually based on hydrofluoric acid (HF) [10–15]. The reaction is catalyzed by metal nanoparticles such as Au [10, 12], Ag [13], or Pt [10, 11] at the substrate surface, and the oxidizing agents are H_2_O_2_ [10–13], KMnO_4_ [14], or Fe(NO_3_)_3_ [15]. It has been shown that SiNWs, which were obtained by MACE, are found to possess such remarkable optical properties as visible photoluminescence (PL) [16], extremely low total reflection [17, 18], enhancement of Raman scattering [17, 19–21], coherent anti-Stokes light scattering [22], interband PL [17, 19–21] and efficiency of generation of third harmonics [23] in comparison with the corresponding intensities for c-Si, and sensitivity of visible PL to molecular surroundings [24]. However, HF is rather toxic and may also result in hypocalcemia and hypomagnesemia [25]. Therefore, there is great interest in the study of possible modifications of the MACE method by using environment-friendly chemistry.

Ammonium fluoride (NH_4_F) has long been used for SiO_2_ dissolution. It has been shown that the SiO_2_ etch rate in aqueous NH_4_F solutions depends on the pH and on the NH_4_F concentration [26]. Then, aqueous NH_4_F solution was proposed as an alternative of HF in the electrochemical method of obtaining PSi films [27–29]. Furthermore, the microstructure properties of the fabricated PSi have shown a strong dependency from the pH of the electrolyte. A pebble-like surface structure was formed during anodization at pH 4.5, while nanoporous Si layers were formed at lower pH [27]. Also, the usage of NH_4_F as the etchant allows it to survive the aluminum contact during the etching process, because the aluminum etch rate in 4.5 % ammonium fluoride is five times lower than that of 5 % HF [28, 29]. It is very important because aluminum is usually used as an interconnect layer in sensors [29].

The possibilities of usage of NH_4_F in the MACE process are shown in [30], where n-type Si wafers were covered with sparse Ag particles and then etched in solutions with different NH_4_F concentrations. Examination by scanning electron microscope (SEM) shows that the etching of the smooth surface of n-Si (100) in 1.0 M NH_4_F + 5.0 M H_2_O_2_ for 1 h is leading to the formation of a PSi-like surface, where the pores were 50–150 nm in diameter and 200–300 nm in depth. In contrast, only a few shallow pores on the Si (100) surface could be formed after the etching conducted in 11.0 M NH_4_F + 5.0 M H_2_O_2_ for 1 h [30]. In [31], the Si (100) surfaces, covered with sparse Ag particles, were put in 1.0 M NH_4_F + 5.0 M H_2_O_2_ to investigate their dark etching. It has been shown, that the morphology on the surface etched for 1 h revealed a sparse distribution of nanopores (10~40 nm in diameter) according to the locations of Ag particles. However, it exhibited porous surface consisting of micropores (1.5~3.1 μm in diameter with 15~20 μm in depth) where nanopores (100~150 nm in diameter) were embedded inside for the etching duration prolonged for 5 h. In ref. [32], it has been shown that the replacement of HF on the NH_4_F aqueous solution in the first MACE step, and also the usage of NH_4_F solution for subsequent deposition of silver particles on SiNW’s sidewall, leads to a good coating of SiNWs without formation of silver dendrites and etching pits. Such silver-coated SiNWs are proposed for surface-enhanced Raman scattering application [32]. It was also showed that additional etching of SiNWs in NH_4_F leads to their surface coverage mainly with Si-(O^−^)_*x*_ species (*x* = 1–3) [33]. This, in its turn, leads to the greater stability of the samples, which is important for creating SiNW-based sensors [34].

In this study, SiNW arrays were prepared by MACE method, where HF is changed on NH_4_F in both etching steps: the decoration of the c-Si layer with Ag nanoparticles was done by using of 0.02 M of AgNO_3_ and 5 M of NH_4_F, and the etching was done in the solution containing 5 M of NH_4_F and 30 % of H_2_O_2_. The structure and optical properties (total reflectance, Raman scattering, and PL) of SiNWs are investigated in detail.

## Methods

**Fig. 1:**
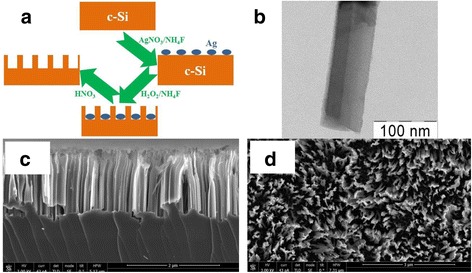
Typical TEM and SEM micrographs of SiNWs. **a** Schematic step-by-step representation of SiNWs preparation method. **b** TEM micrograph of a single SiNW. **c** SEM cross-sectional micrograph of SiNWs. **d** SEM micrograph of SiNWs (view from above)

SiNWs were fabricated by MACE of p-type (100)-oriented c-Si wafer with specific resistivity of 10–20 Ω cm. The difference between the standard MACE method and the new approach was in the changing of HF on NH_4_F. The pH value of the NH_4_F solution was changed to 2.0 by adding H_2_SO_4_ droplets. It was made to have H^+^ ions in the reactions. The pH value was controlled with a calibrated pH meter Checker1 (Hanna Instruments). Prior the MACE procedure, the c-Si substrate was rinsed in 2 % HF solution for 1 min to remove native oxide. Then, in the first step of MACE, thin (~100 nm) layers of Ag nanoparticles of different morphology were deposited on the substrates by immersing them in aqueous solution of 0.02 M of silver nitrate (AgNO_3_) and 5 M of NH_4_F in the volume ratio of 1:1 for 30 s. In the second step, the c-Si substrates covered with Ag nanoparticles were immersed in the solution containing 5 M of NH_4_F and 30 % H_2_O_2_ in the volume ratio of 10:1 in a Teflon vessel for 5 min. All the etching steps were performed at room temperature. Then, the sample were rinsed several times in de-ionized water and dried at room temperature. Finally, SiNW arrays were immersed in concentrated (65 %) nitric acid (HNO_3_) for 15 min to remove residual Ag nanoparticles from the SiNWs. Figure 1a shows the step-by-step formation of SiNWs. The main net etching reaction described in ref. [35] is as follows:

1 $$ \mathrm{S}\mathrm{i}\kern0.5em +\kern0.5em {\mathrm{H}}_2{\mathrm{O}}_2\kern0.5em +\kern0.5em 6{\mathrm{F}}^{-}\kern0.5em +\kern0.5em 4{\mathrm{H}}^{+}\kern0.5em =\kern0.5em \mathrm{S}\mathrm{i}{\mathrm{F}}_6^{2-}\kern0.5em +\kern0.5em 4{\mathrm{H}}_2\mathrm{O}, $$

where ions F^−^ and H^+^ were obtained not from the dissociation of HF as in the standard MACE method but from the dissociation of NH_4_F and H_2_SO_4_. Ag was a catalyst.

The structure properties of SiNWs were investigated by using a SEM of Lyra3 Tescan and a TEM of LEO912 AB Omega. The total reflectance spectra in the region from 250 to 1500 nm were studied with a Perkin Elmer Lambda 950 spectrometer equipped with an integrating sphere. The interband PL and Raman spectra under excitation with a cw Nd:YAG laser at 1.064 μm (excitation intensity ~100 mW; spot size ~2 mm) were measured in a backscattering geometry with a Fourier transform infrared (FTIR) spectrometer of Bruker IFS 66v/S equipped with a FRA-106 unit. PL of SiNWs in the spectral region of 500–1100 nm under excitation with ultraviolet (UV) radiation of an N_2_ laser (wavelength 337 nm, pulse duration 10 ns, repetition rate 100 Hz) was detected by using a grating spectrometer (MS 750, SOLAR TII) equipped with a CCD unit. All measurements were carried out at room temperature in air.

## Results and Discussion

Figure 1b shows a TEM micrograph of a single SiNW. The diameter of the SiNW is about 80 nm, and it is nearly constant through the whole SiNW length. The sidewall surface of the SiNW is porous and contains silicon nanocrystals with the diameter of a few nanometers. Thus, SiNWs are structured as a single-crystal core that replicates the crystallographic orientation of the substrate and are covered by a thin (<10 nm) nanostructured layer. The same structure of SiNWs was observed for SiNWs fabricated on the lightly doped (10 Ω cm) substrate by standard MACE method [24].

A typical large-scale cross-sectional SEM micrograph of a sample with SiNWs is shown in Fig. 1c. One can see that SiNWs look like quasi-ordered arrays with preferential orientation along the [100] crystallographic direction. The length of SiNW arrays is about 2 μm. From the SEM micrograph (view from above) is seen the high density of SiNW arrays (Fig. 1d).

**Fig. 2:**
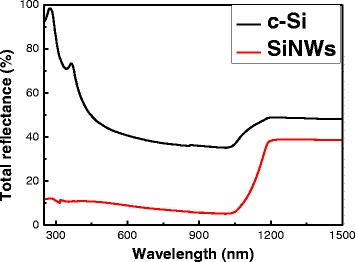
Total reflectance spectra. Total reflectance spectra of c-Si (*black curve*) and SiNWs (*red curve*)

Figure 2 shows the total reflectance spectra of SiNWs and the c-Si substrate. The total reflectance of c-Si demonstrates the well-known behavior of the wafer, the reflection value of ~30 % for the range of the strong absorption (*λ* < 1 μm), and for the transparency region (*λ* > 1 μm), the reflection value increases because of both side contributions. SiNWs exhibit a strong decrease of the total reflectance to 5–15 % in the spectral region *λ* < 1 μm in comparison to the c-Si substrate. In the visible spectral region, the sample with SiNWs looks similar to “black silicon” and can be used as antireflection coating in photovoltaic applications. The same property was observed for samples with SiNWs fabricated by standard MACE method [17, 18], and it can be explained by the strong light scattering and absorption, which results in partial localization (trapping) of the excitation light in SiNW arrays.

**Fig. 3:**
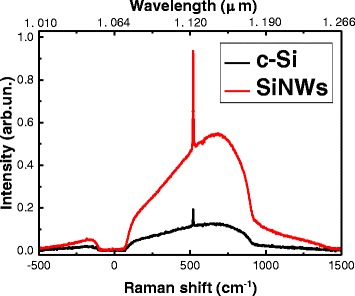
Interband photoluminescence and Raman spectra. Interband photoluminescence (broadband) and Raman spectra (sharp peak at 520 cm^−1^) of c-Si (*black curve*) and SiNWs (*red curve*)

The effect of light localization can be confirmed by analyzing the Raman spectra of SiNWs. Figure 3 shows the typical spectra of the interband PL (broadband) and Raman scattering (sharp peak at 520 cm^−1^) of SiNWs and c-Si substrate for comparison. The PL band and Raman peak position and shape for SiNWs are similar to the c-Si substrate because the SiNW’s diameter is about 50–200 nm and it is far from the quantum confinement regime. The PL and Raman intensities increase strongly for the SiNWs in comparison with the corresponding value of the c-Si substrate. The same results were observed for SiNWs fabricated by standard MACE method [17, 19–21] and can be interpreted as an increase of the excitation intensity of SiNWs because of the strong light scattering and the partial light localization in inhomogeneous optical medium.

**Fig. 4:**
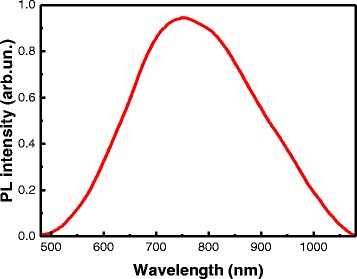
Photoluminescence spectra. PL in the spectral region of 500–1100 nm of SiNWs

Along with the interband PL, the PL of SiNWs in the spectral region of 500–1100 nm with maximum at 750 nm (photon energy of 1.65 eV) was also detected, as it is shown in Fig. 4. This PL can be explained by the radiative recombination of excitons in small Si nanocrystals at the nanowire sidewalls (Fig. 1b) in terms of a quantum confinement model. The nanocrystal size was estimated from the spectral position of the PL peak by using the equation [36]

2 $$ h{v}_{\mathrm{PL}}\kern0.5em =\kern0.5em {E}_g\kern0.5em +\kern0.5em \frac{3.73}{d^{1.39}}, $$

where *hv*_PL_—PL peak position (eV), *E*_*g*_—band gap of c-Si (1.12 eV), and *d*—nanocrystal size (nm). With the photon energy of 1.65 eV, the size of nanocrystals was about 4 nm. Same nanocrystals were observed on nanowire sidewalls in [18, 24]. The presence of the PL in the spectral region of 500–1100 nm will allow the use of SiNWs as luminescent labels for living cells as was shown for SiNWs fabricated by the standard MACE method [18].

## Conclusions

Total reflectance, interband PL, Raman scattering, and PL in the spectral region of 500–1100 nm were investigated. SiNWs exhibit a strong decrease of the total reflectance to 5–15 % in the spectral region *λ* < 1 μm in comparison to the c-Si substrate. The PL and Raman intensities increase strongly for the SiNWs in comparison with the corresponding value of the c-Si substrate. The nanocrystal size at nanowire sidewalls was estimated from the spectral position of the PL and was about 4 nm. It was shown that optical properties of SiNWs formed by MACE using NH_4_F were not much different from optical properties of SiNWs formed by the standard MACE technique with HF. It gives an opportunity to obtain high-quality SiNWs for various applications in photonics, photovoltaics, and sensorics using environment-friendly chemistry.

## Abbreviations

c-Si, crystalline silicon; FTIR, Fourier transform infrared; MACE, metal-assisted chemical etching; PL, photoluminescence; PSi, porous silicon; SEM, scanning electron microscope; SiNWs, silicon nanowires; TEM, transmission electron microscope; UV, ultraviolet

## References

[1] Yang P, Yan R, Fardy M (2010). Semiconductor nanowire: what’s next?. Nano Lett.

[2] Föll H, Hartz H, Ossei-Wusu E, Carstensen J, Riemenschneider O (2010). Si nanowire arrays as anodes in Li ion batteries. Phys Status Solidi RRL.

[3] Bronstrup G, Jahr N, Leiterer C, Csaki A, Fritzsche W, Christiansen S (2010). Optical properties of individual silicon nanowires for photonic devices. ACS Nano.

[4] Kelzenberg MD, Turner-Evans DB, Kayes BM, Filler MA, Putnam MC, Lewis NS, Atwater HA (2008). Photovoltaic measurements in single-nanowire silicon solar cells. Nano Lett.

[5] Sivakov V, Andrä G, Gawlik A, Berger A, Plentz J, Falk F, Christiansen SH (2009). Silicon nanowire-based solar cells on glass: synthesis, optical properties, and cell parameters. Nano Lett.

[6] Cui Y, Wei Q, Park H, Lieber CM (2001). Nanowire nanosensors for highly sensitive and selective detection of biological and chemical species. Science.

[7] Wang X, Ozkan CS (2008). Multisegment nanowire sensors for the detection of DNA molecules. Nano Lett.

[8] Cao A, Sudhölter EJ, de Smet LC (2015). Silicon nanowire‐based devices for gas-phase sensing. Sensors.

[9] Wagner RS, Ellis WC (1964). Vapor–liquid–solid mechanism of single crystal growth. Appl Phys Lett.

[10] Li X, Bohn PW (2000). Metal-assisted chemical etching in HF/H_2_O_2_ produces porous silicon. Appl Phys Lett.

[11] Chattopadhyay S, Li X, Bohn PW (2002). In-plane control of morphology and tunable photoluminescence in porous silicon produced by metal-assisted electroless chemical etching. J Appl Phys.

[12] Dawood MK, Tripathy S, Dolmanan SB, Ng TH, Tan H, Lam J (2012). Influence of catalytic gold and silver metal nanoparticles on structural, optical, and vibrational properties of silicon nanowires synthesized by metal-assisted chemical etching. J Appl Phys.

[13] Sivakov VA, Bronstrup G, Pecz B, Berger A, Radnoczi GZ, Krause M, Christiansen SH (2010). Realization of vertical and zigzag single crystalline silicon nanowire architectures. J Phys Chem C.

[14] Bai F, Li M, Huang R, Song D, Jiang B, Li Y (2012). Template-free fabrication of silicon micropillar/nanowire composite structure by one-step etching. Nanosc Res Lett.

[15] Nahidi M, Kolasinski KW (2006). Effects of stain etchant composition on the photoluminescence and morphology of porous silicon. J Electrochem Soc.

[16] Sivakov VA, Voigt F, Berger A, Bauer G, Christiansen SH (2010). Roughness of silicon nanowire sidewalls and room temperature photoluminescence. Phys Ref B.

[17] Osminkina LA, Gonchar KA, Marshov VS, Bunkov KV, Petrov DV, Golovan LA, Sivakov VA, Timoshenko VY (2012). Optical properties of silicon nanowire arrays formed by metal-assisted chemical etching: evidences for light localization effect. Nanosc Res Lett.

[18] Gonchar KA, Osminkina LA, Galkin RA, Gongalsky MB, Marshov VS, Timoshenko VY, Kulmas MN, Solovyev VV, Kudryavtsev AA, Sivakov VA (2012). Growth, structure and optical properties of silicon nanowires formed by metal-assisted chemical etching. J Nanoelectr Optoelectr.

[19] Gonchar KA, Golovan LA, Timoshenko VY, Sivakov VA, Christiansen S (2010). Effects of light localization in photoluminescence and Raman scattering in silicon nanostructures. Bull Russ Acad Sci Phys.

[20] Timoshenko VY, Gonchar KA, Golovan LA, Efimova AI, Sivakov VA, Dellith A, Christiansen SH (2011). Photoluminescence and Raman scattering in arrays of silicon nanowires. J Nanoelectr Optoelectr.

[21] Gonchar KA, Osminkina LA, Sivakov V, Lysenko V, Timoshenko VY (2014). Optical properties of nanowire structures produced by the metal-assisted chemical etching of lightly doped silicon crystal wafers. Semiconductors.

[22] Golovan LA, Gonchar KA, Osminkina LA, Timoshenko VY, Petrov GI, Yakovlev VV (2012). Coherent anti-Stokes Raman scattering in silicon nanowire ensembles. Laser Phys Lett.

[23] Zabotnov SV, Kholodov MM, Georgobiani VA, Presnov DE, Golovan LA, Kashkarov PK (2016). Photon lifetime correlated increase of Raman scattering and third-harmonic generation in silicon nanowire arrays. Laser Phys Lett.

[24] Georgobiani VA, Gonchar KA, Osminkina LA, Timoshenko VY (2015). Structural and photoluminescent properties of nanowires formed by the metal-assisted chemical etching of monocrystalline silicon with different doping level. Semiconductors.

[25] Bertolini JC (1992). Hydrofluoric acid: a review of toxicity. J Emerg Med.

[26] Judge JS (1971). A study of the dissolution of SiO_2_ in acidic fluoride solutions. J Electrochem Soc.

[27] Dittrich T, Rauscher S, Timoshenko VY, Rappich J, Sieber I, Flietner H, Lewerenz HJ (1995). Ultrathin luminescent nanoporous silicon on n-Si: pH dependent preparation in aqueous NH_4_F solutions. Appl Phys Lett.

[28] Kuhl M, O’Halloran GM, Gennissen PTJ, French PJ (1998). Formation of porous silicon using an ammonium fluoride based electrolyte for application as a sacrificial layer. J Micromech Microeng.

[29] Ohji H, French PJ (1999). Single step electrochemical etching in ammonium fluoride. Sens Actuat A Phys.

[30] Chuang CL, Lin JC, Chao KH, Lin CC, Lerondel G (2012). On wet etching of n-Si (100) coated with sparse Ag-particles in aqueous NH_4_F with the aid of H_2_O_2_. J Electrochem Sci.

[31] Lin JC, Chuang CL, Lin CC, Lerondel G (2012). Development of micro-pores including nano-pores on n-Si (100) coated with sparse Ag under dark etching in 1.0 M NH_4_F containing 5.0 M H_2_O_2_. J Electrochem Sci.

[32] Sun X, Lin L, Li Z, Zhang Z, Feng J (2009). Fabrication of silver-coated silicon nanowire arrays for surface-enhanced Raman scattering by galvanic displacement processes. Appl Surf Sc.

[33] Chen WW, Sun XH, Wang SD, Lee ST, Teo BK (2005). Etching behavior of silicon nanowires with HF and NH_4_F and surface characterization by attenuated total reflection Fourier transform infrared spectroscopy: similarities and differences between one-dimensional and two-dimensional silicon surfaces. J Phys Chem B.

[34] Masood MN, Carlen ET, van den Berg A (2014). All-(111) surface silicon nanowire field effect transistor devices: effects of surface preparations. Mat Sci Semicond Process.

[35] Zhang M-L, Peng K-Q, Fan X, Jie J-S, Zhang R-Q, Lee S-T, Wong N-B (2008). Preparation of large-area uniform silicon nanowires arrays through metal-assisted chemical etching. J Phys Chem C.

[36] Ledoux G, Guillois O, Porterat D, Reynaud C, Huisken F, Kohn B, Paillard V (2000). Photoluminescence properties of silicon nanocrystals as a function of their size. Phys Rev B.

